# Nano-Enable Materials Promoting Sustainability and Resilience in Modern Agriculture

**DOI:** 10.3390/nano11082068

**Published:** 2021-08-15

**Authors:** Hafeez Ur Rahim, Muhammad Qaswar, Misbah Uddin, Cinzia Giannini, Maria Lidia Herrera, Giuseppina Rea

**Affiliations:** 1 Key Laboratory of Industrial Ecology and Environmental Engineering (Ministry of Education), School of Environmental Science and Technology, Dalian University of Technology, Dalian 116024, China; Hafeez.kalpani@aup.edu.pk (H.U.R.); misbahswat@yahoo.com (M.U.); 2 Department of Environment, Ghent University, Coupure Links 653, 9000 Ghent, Belgium; mqaswar2@gmail.com; 3 Institute of Crystallography, CNR, Via Amendola 122/O, 70126 Bari, Italy; cinzia.giannini@ic.cnr.it; 4 Institute of Polymer Technology and Nanotechnology, Facultad de Arquitectura, University of Buenos Aires-CONICET, Diseño y Urbanismo, Intendente Güiraldes 2160, Pabellón III, Ciudad Autónoma de Buenos Aires C1428EGA, Argentina; mlidiaherrera@gmail.com; 5 Institute of Crystallography, CNR, Via Salaria Km 29,300, 00015 Roma, Italy

**Keywords:** food security, food safety, nano-agrochemicals, nanosensors, smart-packaging, sustainable development goals

## Abstract

Intensive conventional agriculture and climate change have induced severe ecological damages and threatened global food security, claiming a reorientation of agricultural management and public policies towards a more sustainable development model. In this context, nanomaterials promise to support this transition by promoting mitigation, enhancing productivity, and reducing contamination. This review gathers recent research innovations on smart nanoformulations and delivery systems improving crop protection and plant nutrition, nanoremediation strategies for contaminated soils, nanosensors for plant health and food quality and safety monitoring, and nanomaterials as smart food-packaging. It also highlights the impact of engineered nanomaterials on soil microbial communities, and potential environmental risks, along with future research directions. Although large-scale production and in-field testing of nano-agrochemicals are still ongoing, the collected information indicates improvements in uptake, use efficiency, targeted delivery of the active ingredients, and reduction of leaching and pollution. Nanoremediation seems to have a low negative impact on microbial communities while promoting biodiversity. Nanosensors enable high-resolution crop monitoring and sustainable management of the resources, while nano-packaging confers catalytic, antimicrobial, and barrier properties, preserving food safety and preventing food waste. Though, the application of nanomaterials to the agri-food sector requires a specific risk assessment supporting proper regulations and public acceptance.

## 1. Introduction

In the past 50 years, conventional farming systems have gained a tremendous improvement in productivity and efficiency, leading to substantial increases, ranging from 70% to 90%, in food availability and affordability [1].

However, some conventional agricultural practices, including large-scale cultivation of monoculture, exploitation of highly productive hybrid crops, extensive use of enhancers (water, pesticides, and fertilizers), and high stock density grazing livestock, raise various ecological, economic, and social concerns that require a transition to more sustainable agriculture and food practices [2].

Ecological concerns entail the gradual erosion of exposed topsoil (desertification) due to loss of organic matter and structure, soil compaction, and salinization, leading to a decline of fertile land and consequently soil productivity. Furthermore, the intensive exploitation of mineral fertilizers, mainly nitrates and phosphorus, but also organic fertilizers, including manure, leads to an increase in greenhouse gas emissions, eutrophication of inland, ground, and marine waters resulting in algae blooms, fish kill, and public health threats [3]. Similarly, the massive application of synthetic and even bio-pesticides or antibiotics results in soil, water, and food contamination, despite poor effectiveness due to low efficiency of delivery and utilization.

These events pose serious health hazards to consumers and farmworkers at high risk of exposure and lead to the emergence of pesticide resistance in pathogens as well as in beneficial species (e.g., pollinators or soil microorganisms), seriously harming the ecosystems and, consequently, determining food shortage. Meanwhile, the ongoing climate changes fostered by agricultural practices could enhance the degradation of non-renewable resources, further impairing crop yields and agricultural production [4].

However, as the present global population is estimated to grow up to 9.6 billion by 2050 and to 11.2 billion by 2100, at least a 50% increase in agri-food production will be needed to guarantee food security according to the 2030 agenda of sustainable development goals (SDGs) [5]. Therein, the United Nations (UNs) and Food and Agriculture Organization (FAO) pledged themselves to reduce hunger and poverty and to ensure food security by building sustainability and resilience in the agricultural system [6].

Sustainable agriculture should be thinking up a multi-tiered framework embracing economic, societal, and ecological factors, aiming to address present needs without compromising those future generations. Achieving sustainable agriculture implies not only preserving biodiversity, land, soil, and environmental health but also improve the quality and quantity of agricultural products, develop smart management, exploitation, and consumption of resources and energy. These create profits for investors and farmers promoting health and advancing social conditions and develop a fast-responsive versatile system capable of adjusting to climate change.

In this context, the application of innovative technologies lowering the environmental footprint of agriculture could certainly be decisive, and according to recent findings, nanotechnology bears enormous problem-solving capability [7].

Research on nanotechnology deals with fabrication, manipulation, and characterization of matter with characteristic dimensions in the nanoscale range (1–100 nm) in at least one dimension. At this length scale, materials acquire distinctive shapes and physico-chemical properties compared to their bulk counterpart [8], and the novel acquired structural, optical, chemical, electrical, or magnetic features could be exploited to build sustainability. Indeed, nanotechnology is regarded as a key enabling technology, having a strong impact on scientific innovation, economy, and society, including industrial competitiveness, creation of wealth, and quality of life, and already find application in electronics, engineering, and medicine [9]. More recently, a great potential has also been envisaged in the agri-food industry, where nanoscale materials, namely engineered nanomaterials (ENMs), are emerging as efficient countermeasures to the conventional agriculture-induced shortcomings fostering food safety and security [10].

This review gathers recent research innovations on smart nanoformulations and delivery systems improving crop protection and plant nutrition, nanoremediation strategies for contaminated soils, nanosensors for plant health and food quality and safety monitoring, and nanomaterial related solutions for smart food packaging. It also highlights the impact of engineered nanomaterials on soil microbial communities and potential environmental and human health risks. Future research directions are also proposed fostering an efficient, sustainable, resilient, nanotechnology-based agricultural system to meet the 2030 agenda of SDGs about agriculture, environment, and food security.

## 2. Improving Crop Production via Nanotechnology-Enabled Innovations

The awareness that conventional farming technologies would not increase crop production on a sustainable basis motivated research on nanotechnology application in agriculture, revealing positive effects of ENMs on plant growth and development, seed priming and germination, detection of pathogens and contaminants, plant nutrition and protection from biotic and abiotic stresses, soil fertilization, microflora protection, remediation of contaminated soils, food science, and many others [11].

**Figure 1:**
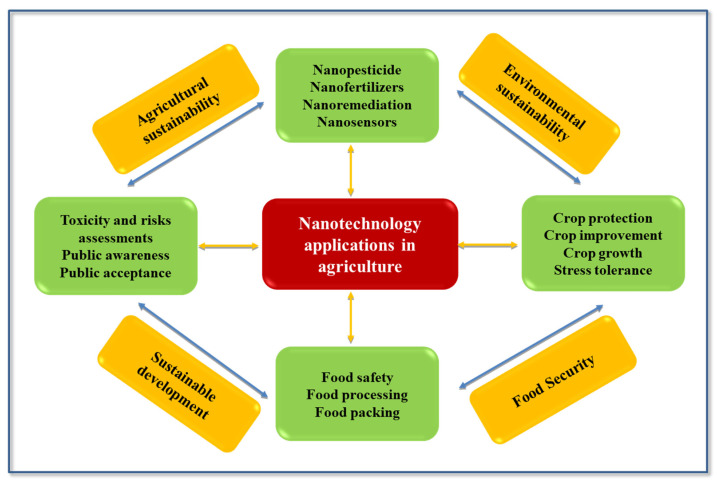
Nanotechnology applications in agriculture. Nanopesticides and nanofertilizers provide targeted and controlled release of active ingredients (AI), reducing environmental impact while sustaining plant protection from disease and crop yield, respectively; ENMs for soil remediation are regarded as more efficient tools for polluted soils clean-up; nanosensor devices are suitable for monitoring either plant stress or food quality and safety. Similarly, they play a role in food processing and packaging.

ENMs are, hence, expected to enhance sustainability and building climate resilience to achieve the 2030 SDG. However, as in any new technology, it is mandatory to rigorously address risk/benefit issues by assessing the potential toxicity of nanotechnology applications before any authorization of commercialization. For some already-in-use ENMs, their applications and outcomes are schematically depicted in Figure 1 and will be discussed in the following sections.

### 2.1. Nanotechnology-Based Agrochemicals for Sustainable Agriculture and Food Systems

Over 3 billion tons of a wide variety of crops are produced annually throughout the world for human consumption (wheat, rice, legumes, etc.), livestock (maize, etc.), fuel, textile, and other uses, requiring high amounts of pesticides, fertilizers, water, and energy [12]. Agrochemicals are hence regularly used to protect crops from various pests to ensure food security and safety. However, most agrochemicals have very low use efficacy, and average losses range from 10% to 75%, depending on the application mode, e.g., foliar or soil application; physical/chemical properties, e.g., solubility and weak acidic properties modulating uptake and translocation, and retention to the soil. These drawbacks stimulated intense research in the development of innovative nanoscale formulations, likely due to their distinctive properties: small dimension enabling a more efficient penetration in the plant cells, high volume surface ratio enabling increased loading of the AI, high tunability enabling the development of targeted delivery [13,14].

#### 2.1.1. Nano-Enabled Pesticides for Plant Protection

Pesticides are a heterogeneous class of chemical compounds, including, among others, insecticides, fungicides, acaricides, and virucides. Their application is indispensable in crop production, but due to the high-dose supply, bioaccumulation through the food chain has emerged, posing dangerous risks to wildlife and humans. Furthermore, the effectiveness of some synthetic pesticides is restricted by their lability and retention in the soil organic matter to stave off their accumulation at the root level, where many pests reside, hence affecting their function. In addition, excess pesticides could leach from farm soils and move away from the site of application triggering environmental pollution [15]. In this context, research efforts focused on the design and generation of novel classes of smart nanopesticides having improved efficacy, stability, and effective duration, while reducing their enormous pressure on biodiversity and related environmental and human health. The term nanopesticides is generally referred to nanoscale slow delivery systems aimed to replace conventional pesticides consisting of encapsulated organic or inorganic AI, which are exploited in either conventional or organic agriculture [16].

These systems rely on the utilization of nanomaterials as carriers for the adsorption, encapsulations, or conjugation of classical pesticides to enhance stability, biodegradability, permeability, dispersion, and delivery of the pesticide [17]. The rationale behind the use of these nanocarrier formulations relies upon their size-specific properties ranging between 10 and 200 nm, which justifies the considerable surface-volume ratio that determines: (1) increased interactions of nanopesticides with targeted pests even at low dose applications; (2) reduction of pesticides waste; (3) enhancement of water retention; (4) improvement of water-solubility [18].

**Figure 2:**
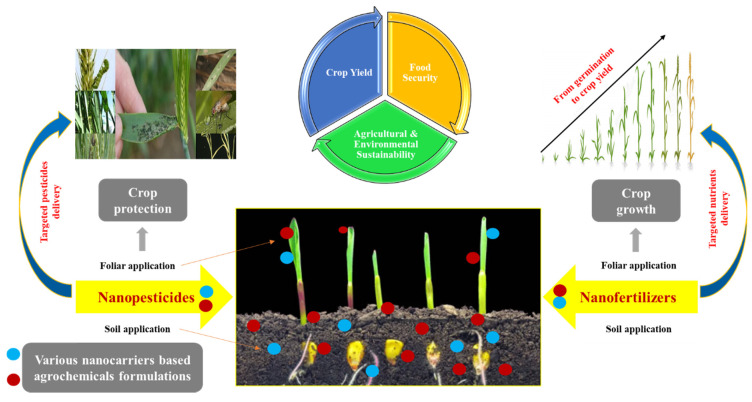
A schematic diagram illustrating the application of nanopesticides and fertilizers in modern agriculture. The targeted delivery of these nano-agrochemicals via foliar and/or soil application provides protection to crops from various pests and enhances utilization and uptake efficiency of nutrients for ameliorating crop growth and yield in order to achieve food security, agricultural, and environmental sustainability.

As stated above, nanopesticides showed advantages over classical pesticides, such as low volume usage, high efficiency, and high deposition on plant leaves due to the small size. Classical pesticides have coarse drug carrier particles, poor dispersibility, poor stability, and low biological activity. Moreover, their utilization rate for targeted crops is less than 30% [19]. Therefore, to achieve successful crop protection, attain maximum crop yield, ensure food security, sustainability, and resilience, researchers have recently investigated novel nanocarriers and formulations for delivery of the targeted pesticides as illustrated in Figure 2, including mesoporous silicon, polymeric nano-capsules, and cyclodextrin polymers, which are presented below.

Among the commercially available nanopesticides, copper (Cu)-based nanoparticles (NPs) are massively produced and find applications not only in agriculture [20] but also in food preservation and water treatments. They are widely used in agriculture due to the antimicrobial and antifungal properties of Cu ions that enable the control of a plethora of pathogens.

As an example, the Cu-based fungicides and bactericides Kocide^®^3000, one of the most applied pesticides on trees and crops to destroy blight, black spot, and downy mildew, contains nanosheets composed of nanoneedles of copper(II) hydroxide, Cu(OH)_2_, and it is extensively explored in terms of ecological impacts [21]. In these studies, the impact of a commercial Cu(OH)_2_ nanopesticide formulation on bacterial metabolic activity and community structure of loamy soil in comparison with its ionic analog (CuSO_4_) and nano-Cu(OH)_2_ was investigated. As a whole, the impacts of nano-Cu(OH)_2_ on soil bacterial community and enzyme activity differed from its ionic analog, showing the environmental risks of nano-Cu(OH)_2_ nanopesticides in the long term [22]. Another informative study provided insights on a commercially available colloid-size Cu(OH)_2_ fungicide/bactericide highlighting the influence of elemental composition in addition to physico-chemical properties on the fate, transport, stability, dispersion, and dissolution of the nanoformulations [23]. In this context, the relevance of risk assessment evaluation either on the AI or the additive should be emphasized, as the latter could also result in unintended consequences [24].

A slow-release formulation of the herbicide mesotrione (MS) was designed by incorporating the herbicide in micelles formed by the fatty amine ethoxylated surfactant Ethomeen T/15 followed by their further sorption on the clay mineral sepiolite for extended weed control. The results highlighted that the utilization of designed sepiolite-based formulation significantly reduced the leaching of MS, compared to a conventional formulation (i.e., percentage of the total amount extracted varied from 16.3 ± 3.1% for designed sepiolite-based formulation to 26.3 ± 3.4% for the conventional formulation, 30 days after being applied), while keeping the desired bioactivity for weed control. Moreover, no significant differences in soil accumulation at longer times and in crop yield were observed [25].

In the class of electro-responsively controlled-release systems, a novel electrical-driven release and migration herbicide glyphosate (Gly) was fabricated by using attapulgite (ATP, a fibrous clay mineral made by a magnesium-aluminum phyllosilicate), calcium alginate (CA), and Gly. Herein, the ATP-CA acted as a network-structured carrier to efficiently bind Gly, giving rise to ATP-GLY-CA porous hydrogel spheres via a crosslinking reaction. Under a specific electrical field, the CA pores were enlarged, promoting the efficient and precise release and migration of Gly. According to the authors, this system is low-cost and might have high application value by enhancing the utilization efficiency of Gly [26].

Biodegradable hydrogels (BHGs), such as edible polymers, natural gums, natural fibers, synthetic polymers, chitosan, cellulose, starch, lignin, gelatin, alginate, biochar, pectin, and clay and their derivatives have been studied because of their biocompatibility, biodegradability, low toxicity, and chemical alteration. Due to their unique properties, the regenerative nature of BHGs’ has innovative applications in various fields, including wastewater treatment, control, restoration, and agricultural applications as agrochemical carriers for their gradual or continuous release, as well as other value-added applications [27].

The successful fabrication of biochar-based hydrogel microspheres was developed to prepare a pH and ion strength dual-stimuli responsive controlled-release system for hydrophilic pesticides (gentian violet). This work provided a simple and low-cost approach and preparation procedure that could regulate pesticide release behavior, reduce leaching losses, and enhance the utilization efficiency of pesticides [28]. Biochar prepared from rice husk (rBC) was used as a nanosorbent for the sustained delivery of 2,4-dichlorophenoxyacetic acid (2,4-D) and for its potential use as an eco-friendly nanoherbicide formulation (DrBC). The release profile of 2,4-D from DrBC was found to be a controlled diffusion mechanism based on Korsmeyer–Peppas model fit. This research study presented a green herbicidal formulation, a promising herbicidal loading, sustained release, and reduced leaching of 2,4-D [29].

Xie and co-workers developed an environmental-friendly controlled release system for the insecticide spirotetramat in an alginate matrix. Four formulations, starch–chitosan–calcium alginate (SCCA), starch–calcium alginate (SCA), chitosan–calcium alginate (CCA), and calcium alginate (CA) complex gel beads, were prepared by the extrusion–exogenous gelation method. The results showed the release behaviors of the formulations in water could be well described by the logistic model, and the release occurred through Fickian diffusion. Among the four formulations, SCCA showed the highest entrapment efficiency, drug loading, and the slowest release rate. This work provided a possible approach to prolong the shelf-life of spirotetramat and reduce environmental contamination [30]. In another study, the successful fabrication of carboxymethyl chitosan (CMCS) based nanopesticides with controlled-release properties was demonstrated. A trisiloxane surfactant (TSS) was used to improve the CMCS hydrophobicity through an intermediated grafting reaction with allyl glycidyl ether (AGE). The resulting CMCS-AGE-TSS was used to encapsulate by self-assembling the anthelmintic and insecticidal pesticide Avermectin (AVM), obtaining the AGE-TSS@AVM NPs. The latter demonstrated a better spreadability and adherence to the leaves, slow-release under neutral conditions, increased shelf-life (from 45 to 83 min), and similar insecticidal activity compared to pure AVM [31].

The development, feasibility, and potential of the flash nanoprecipitation (FNP) method in the universal fabrication of multifunctional NPs with in situ pesticide tracing and crop protection capabilities was discussed by Chen and co-workers [32]. Among the numerous preparation strategies, FNP has several advantages, including a quick and continuous assembly process, efficient encapsulation rate, and controllable particle size [33]. The authors generated three types of NPs composed of Nile Red (NR) and λ-Cyhalothrin (LC), NR-loaded NPs (NR NPs), LC-loaded NPs (LC NPs), and NPs loaded with NR and LC as co-cores (NR LC NPs) through the FNP method under the same experimental conditions. Fluorescence measurements were used to reveal the internal aggregation state and rearrangement of molecules in the NPs. High-resolution IVIS imaging and confocal laser scanning microscopy analysis showed the deposition and distribution of NPs on leaves could be visualized while avoiding undesirable autofluorescence [32]. Similarly, the formulation of abamectin nanopesticide having extended photostability and sustained release was realized by using the continuous and scalable FNP technique. Economic and biocompatible stabilizers, hypromellose acetate succinate and lecithin, were used in formulations with optimized mass ratios for nanoparticle stability. The optimized formulations not only showed improved photostability compared to free abamectin but also obtained more sustained release behaviors in a controlled manner [34]. FNP is a novel and promising approach for the preparation of bifunctional nanopesticides, which may be extended to other pesticides and fluorescence dyes to achieve superior formulations and fulfill massive potential applications in plant science [32].

It is worth mentioning that nanoencapsulation is also of great relevance for entrapping and delivering natural bioactive compounds, including, for example, essential oils (EOs), which play crucial roles in crop protection. EOs are highly complex mixtures of compounds, comprising mainly mono- and sesqui-terpenes, and phenols, among others, produced by the secondary metabolism of plants as defense responses to biotic stress. EOs, in fact, have antibacterial, antiviral, antifungal, and insecticidal properties, and for these reasons, they have been extensively used to protect stored commodities or to repel pests from human habitations [35]. However, the bioavailability, bioactivity, stability, and solubility of these compounds could be strongly affected during processing and storage, limiting their application. In this context, the adoption of electrohydrodynamic processes, namely electrospinning and electrospraying, represent novel technological opportunities to produce nanofibers and NPs, respectively, which have large surface-to-volume ratios, small pore sizes, and high porosity. In comparison with other techniques, e.g., spray-drying, electrohydrodynamic processes are ideal for encapsulating EOs using mild synthesis conditions, preserving their activity during processing, and promoting controlled delivery [36].

In Table 1, some recent achievements on nanopesticides have been summarized.

#### 2.1.2. Nano-Enabled Fertilizers for Plant Nutrition

Nutrients play a very important role in sustaining soil fertility and increasing plant nutrition for food security and safety. However, similarly to pesticides, largely they are of synthetic origin, have limited nutrients utilization efficiency and present leaching of the AI posing serious threats to the ecosystem and human health [42]. Hence, also in the field of fertilizers, the design of nanotechnology-based fertilizers has been suggested as a valuable tool to overcome the weakness of bulk fertilizers. In the beginning, the effort was the development of novel nano-based fertilizers and formulations to achieve maximum crop yield [43]. Later on, research focused on the development of nanofertilizers aimed to reduce losses of mobile nutrients and improve the accessibility of poorly available nutrients [14].

Nanofertilizers have been shown to improve crop yield and quality with improved nutrient use efficiency and reduced production costs [44]. The advantages of nanofertilizers over sole inorganic fertilizers rely on their slow-release targeted delivery system associated with cementing nanomaterials (Figure 2) [45].

Raliya and co-workers highlighted the current state and future perspectives of nanofertilizers for precision and sustainable agriculture, taking into consideration the type of NPs (TiO_2_, ZnO, MgO, carbon-based, etc.), nanoscale properties, nanoparticle delivery systems, tested plants/crops, and physiological responses [46]. However, there is still a concern about the intentional utilization of NPs for crop cultivation, as residual nanomaterials in crops and the environment will eventually increase their exposure routes, causing possible bioaccumulation through the food chain and potential negative effects on human health. A case study was provided by Servin and collaborators revealing, the application of TiO_2_ NPs in the soil can accumulate in cucumber fruits and potentially lead to bio-amplification in humans [47].

In the literature related to nanotechnology-based applications in agriculture, the terms nanofertilizer and slow-release fertilizers are used in the same context. Therein, in recent years, researchers have suggested the use of controlled-release fertilizers (CRFs) or slow-release fertilizers (SRFs) or controlled release nanomaterials (CRMs) as a new strategy to overcome limitations of the bulk counterpart [48]. Conventional fertilizers display a limited utilization efficiency ranging from 30% to 50%. Furthermore, as the soil applications of these fertilizers are mostly in salt form, they are susceptible to losses via a variety of pathways leading to eutrophication, expenditure increases, and reduced benefits for farmers [49]. Excessive fertilization, incorrect dosage or type of fertilizers can lead to accumulation of salts and, consequently, increased acidification or salinity of soils with risk of heavy metals contamination [50]. For these reasons, it is more worthy of supplying these conventional fertilizers/nutrients to the soil in a precise manner, limiting losses and ecological damage [51].

CRFs possess the novel property of slow-release of their active component over a prolonged period to sustain an adequate delivery and long-term effectiveness for plant growth [52]. To achieve these goals, the AI can be: (i) encapsulated directly inside nanomaterials, such as nanotubes or nanoporous materials; (ii) coated with a thin protective polymer layer; (iii) delivered as a particle or emulsions of nanoscale dimensions [53]. According to Davidson and co-workers, CRFs should enhance the soil release kinetics of chemical fertilizers to avoid or limit losses of fertilizer and environmental damages [54]. In Table 2, we report novel nanofertilizers that, in our opinion, have a high potential to promote plant growth and crop yield.

However, besides the potential of CRFs to enhance crop yield, their occurrence in the market is still limited, mainly due to their actual high costs and raised concerns regarding their effective efficiency. The latter is tightly correlated to the type of coating/polymer protecting the AIs and preventing their pre-mature release caused by soil characteristics. In this context, the exploitation of biopolymers is receiving increasing attention being biodegradable, biocompatible, non-toxic materials, and suitable to improve soil quality [65]. Furthermore, similarly to what was observed for the bulk material, awareness and concerns on possible CRFs-induced soil acidification were raised, calling for a responsible evaluation of risk to avoid land degradation. It should also be taken into consideration soil acidification determines a reduction of the charged cations exchange capacity, hence impairing plant nutrient uptake and CRFs efficacy. Indeed, *in-field* studies on CRFs release pattern, stability, AI uptake, as well as efficacy and possible negative impact to plants and ecosystem require a deeper evaluation.

## 3. Nanoremediation of Contaminated Soils

Soil is a chemically heterogeneous milieu hosting either organic or inorganic matter, as well as living (and not-living) organisms, giving rise to unique ecosystems. Soil is a highly valuable natural resource, but, unfortunately, it is non-renewable, hence requiring careful preservation to avoid unsustainable losses. It is estimated the world’s agricultural land provides about 99% of our food, in addition to make available fibers, wood, and raw materials of industrial interest. It also serves as greenhouse gas regulation, carbon storage, recycling of wastes, control of pathogens, and many others [66]. However, the anthropogenic activities led to a gradual, irreversible degradation of soil quality and health [67]. In parallel, the projected increase in food demand and soil pollution with emerging pollutants places unprecedented pressure on global soils [68]. Pollution derived from natural sources, e.g., volcanoes, can move to spread and vanish in a short time, likely posing limited concerns to human health [69]. On the contrary, contamination derived from anthropogenic activities led to persistent environmental poisoning due to the release of either organic (e.g., bisphenols, plastics, fertilizers, pesticides) or inorganic pollutants, including heavy metals and metalloids (e.g., Cd, Cu, As, Zn, Hg, Pb) [70], as well as new emerging pollutants (e.g., nano/microplastics, pharmaceuticals, personal care products, surfactants, fire retardants, and so on) leading to an ever-increasing concern for of all trophic levels [71].

**Figure 3:**
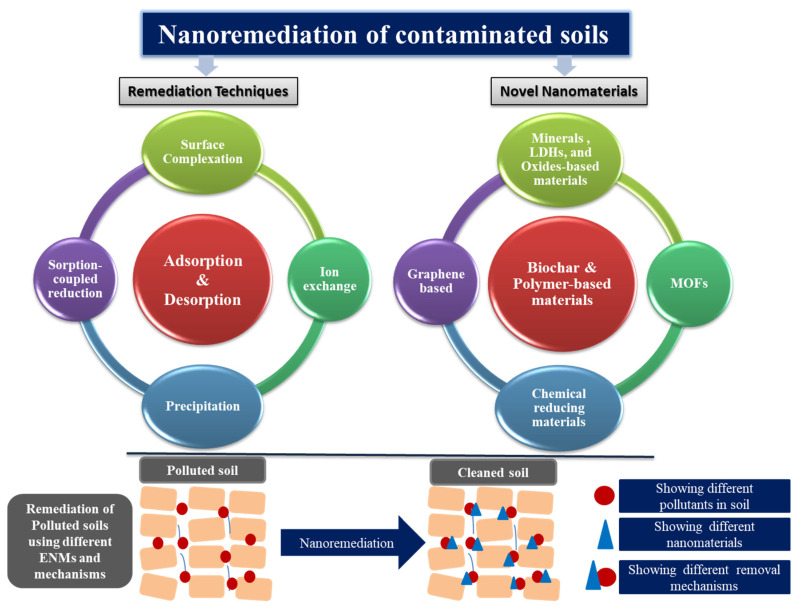
Schematical representation of innovative materials, remediation techniques, and potential removal mechanisms exploited for remediation of polluted soils.

Thereby, the sustainable management of soils and water requires the development of resilient remediation strategies. For decades various treatments techniques, including precipitation, coagulation, flocculation, incineration, ion exchange, reverse osmosis, membrane filtration, electrochemistry, photoelectrochemistry, oxidation process, phytoremediation, and biological techniques have been used for the removal of environmental contaminants [72]. However, the removal and transformation routes of emerging pollutants were different among the different techniques [73]. In the recent past, nano-enabled techniques have opened new routes for reducing pollution poisoning the environment [74]. Among others, adsorption is one of the most exploited, resilient, low-cost, popular, and effective mechanisms to remove emerging pollutants [75]. To this end, advanced nanostructure materials with advantages of unique architectures and preeminent characteristics, such as high specific surface area, well-defined and abundant active sites, and low density, have been developed mainly exploiting carbon-based, graphene-based, metal oxides, minerals, and chemical reductants-based reducing materials (Figure 3). Carbon-based materials including biochar, activated-carbon, single and multi-walled CNTs, and graphene have been found efficient in removing both organic and inorganic contaminants from the environmental components, such as soil, water, and air, with adsorption efficiencies reaching 80% and degradation efficiencies up to 99% [76]. Various metal oxides-based materials, including titanium dioxide (TiO_2_), nanosized manganese oxides (MnOs), iron oxide (FeO), and magnesium oxide (MgO), have also been used extensively to remediate various pollutants in soil [77]. Chemical reductants-based reducing materials, such as based on nano-zerovalent iron (nZVI), iron sulfide (FeS), thiosulfate (S_2_O_3_^−2^), molybdenum sulfides (MoS_2_), manganese (Mn), zinc (Zn), hydrogen peroxide (H_2_O_2_), and others, have the efficiency of transformation, degradation, and detoxification of organic and inorganic pollutants in soils and water. The underlying reaction mechanisms were adsorption, complex formation, immobilization, ion exchange, reduction, S_N_2 nucleophilic substitution, and reductive dehalogenation [78,79,80]. Conclusively, in Table 3, we report recent advances and innovations utilizing nanomaterials for the remediation of contaminated soils, possibly enabling the achievement of the 2030 SDGs of food security, good health and well-being, clean water and sanitation, climate action, and life on land.

## 4. Nanomaterials and Soil Microorganism’s Interactions

As stated above, the applications of ENMs have been used for crop protection and fertilization to maximize crop production, sustainability, and resilience in the food system. However, such benefits could turn into risks for non-target plants, plant-beneficial microbes, and other plant-associated activities of microorganisms when these materials contaminate the environment [91]. Hence, in parallel to positive effects, it is worthy to also address potential or proved negative effects of nanomaterials applied through foliar or soil routes on plant-associated soil microorganisms [92]. However, the literature curation showed a contradictory trend in results and claimed even more in-depth investigations to get insights into the biological conversion and ENPs-plant-soil-microorganisms relationship [93]. Till now, various nano-based particles and materials, including organic and inorganic NPs, metal and metal oxides, CNTs, biochar, and biochar-based nanocomposites, graphene nanomaterials, quantum dots, have been used in the agricultural system whose pathogenic and antimicrobial behavior has been systematically well documented, however, the interactions of nanomaterials with soil microorganisms affecting food production are still poorly explored [94].

Xin et al. (2020) tested the impact of different doses and different NP types on the microbiological and biochemical properties of two different agricultural soils, shading lights on adverse effects of nano-Ag, nano-TiO_2,_ or multi-walled carbon nanotubes (MWCNTs), but not with the newly synthesized polysuccinimide NPs (PSI-NPs) with MWCNTs [95]. Zhang and co-workers analyzed the effects of black phosphorus nanosheets used in environmental remediation as effective adsorbents for ionic organic pollutants, revealing only negligible or short-term effects on enzyme activity and the bacterial community [96].

Furthermore, Biolog EcoPlates were used to investigate the impacts of metal ENMs on bacterial communities in three different soil types. The metabolic fingerprints produced via Biolog EcoPlates presented significant shifts in the presence of Ag and Zn-oxide ENMs, but not in the presence of TiO_2_ [97]. Q. Zhu et al. (2020) reported that nano-maghemite (γ-Fe_2_O_3_) significantly affected malondialdehyde content, reactive oxygen species production, and lactate dehydrogenase activity in the white-rot fungus *P. chrysosporium* cells as an expression of activation of defense mechanisms [98]. Ouyang and colleagues examined the effects of ZnO NPs on the growth and biofilm formation of *Pseudomonas putida* KT2440, a model plant-beneficial bacterium ubiquitously present in the soil, providing evidence on dose-dependent responses leading to efficient nutrient use and biofilm formation at low doses, imbalance of antioxidant systems and inhibition of cell activity at high doses [99]. Mortimer and co-workers [94] deepened the knowledge on previous evidence indicating nanoceria nanoparticle (CeO_2_ NPs), MWCNTs, graphene nanoplatelets (GNPs), and carbon black (CB) inhibit symbiotic nitrogen (N_2_) fixation in soybeans by assessing a direct rhizobial susceptibility. Strong dose-dependent inhibition of growth and remodeling of gene expression impairing nodulation competitiveness has been reported in *Bradyrhizobium diazoefficiens* after exposure to the abovementioned carbon-based NP. Regarding the toxic effect of TiO_2_ on plant growth-promoting soil bacteria (PGPB), Chavan et al. (2020) stated PGPB was significantly constrained when posed in direct contact with TiO_2_ [100]. Liang et al. (2020) evaluated the individual and combined effects of simultaneous application of biochar and compost on the enzyme activity and microbial biomass in wetland soils spiked with the bacteriostatic antibiotic sulfamethoxazole (SMX). After enhancing the physical and chemical properties of soil, dose-dependent effects were observed again in terms of SMX degradation and biomass accumulation [101].

Based on the above literature, we highlight the need for a deeper understanding of the soil microbes/nanomaterials interactions (either positive or negative) and more comprehensive, multi-tiered investigations expanding to different soil communities.

## 5. NanoSensors for Monitoring Pathogens, Diseases, and Environmental Conditions

**Figure 4:**
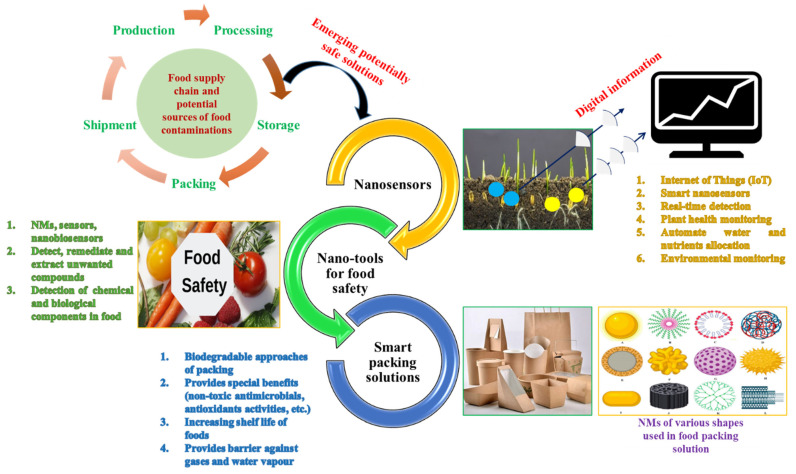
Graphical representation of nanosensors application in agriculture and ENMs in food safety and smart packing. The application of nanosensors, ENMs, and other novel nanotools along with computed-based control systems greatly comes up with more resilient and sustainable food production, processing storage, packing, and transportation systems.

Reliable and timely detection of plant stress plays an important role in crop health monitoring to reduce disease spread and facilitate effective management practices, which in turn helps to prevent food loss, guaranteeing food security. The conventional diagnosis of crop disease includes direct or indirect methods, such as visual inspection of symptoms, serological assays, and DNA-based detection of pathogens. These techniques are efficient but less reliable at the asymptomatic stage, and additionally are time-consuming, expensive in terms of equipment and skilled operator requirements, and cannot be applied in the field [102]. ENMs are emerging as crucial tools to monitor plant health, enabling the construction of smart sensors for the early detection of plant stresses [103]. In the era of the Internet of Things (IoT), smart nanosensors serve as sensing and transducer units of biological signals connecting the living being to digital information. Nanosensors application could enable not only the real-time detection of plant chemical signals for plant health monitoring but also for automated water and nutrient allocation (Figure 4) [104].

Recently, a nanosensor based on single-walled CNTs functionalized with a hemin-decorated DNA aptamer allowing the monitoring of plant health and detection of either biotic or abiotic stresses, was realized. These near-infrared (nIR) fluorescent nanosensors were interfaced with leaves of *Arabidopsis thaliana*, plants enabling the visualization of H_2_O_2_, a key signaling molecule related to the onset of plant stress in remote models. These sensors can find valuable applications for understanding plant-stress communication [105]. The commercial ground-based optical OptRx^TM^ sensor, already used for maize plants, has been evaluated for its capability to monitor the seasonal fertilization requirements of nitrogen (N) in soybean plants, revealing effectiveness later in the season and paving the way for its utilization in soybean plants for crop yield increase [106].

A sensitive nanosensor, based on the fluorescence quenching of green-synthesized carbon dots (CDs), has been optimized to detect and quantify the widely used pesticide DZ. In this study, green CDs were prepared using aqueous and alcoholic extracts of rose flowers with blue, yellow, and red pigments under identical synthesis conditions. The yellowish extract revealed more stability, and it was used to construct the sensors, which exhibited a linear response in the range 0.02–1 µM with a 3.5% relative standard deviation for the detection of 0.01 μM DZ [107]. Similarly, a novel sensitive aptamer-based nanosensor based on the fluorescence properties of reduced graphene quantum dots (rGQDs) and MWCNTs was developed for the rapid detection of diazinon (DZ), one of the most widely used organophosphorus pesticides. The designed apta-nanosensor provided fast response and limit of detection of 0.4 nM (0.1 µg/L) in the linear range of 4–31 nM, complying with the imposed regulations by the European Union (EU) and World Health Organization (WHO). In addition, the sensor revealed high selectivity in real samples, and due to its miniaturized dimension (tap water, urine, river water, and agricultural runoff water), paved the way to its in-field utilization [108]. By the functionalization of activated glassy carbon electrode (GCEox) with graphene quantum dots, chitosan, and nickel molybdate nanocomposites for DZ determination, another sensitive and selective electrochemical sensor was realized. The effect of interfering compounds was studied on the sensor’s function, accompanied by recovery analysis of DZ in cucumber and tomato real samples [109]. A fluorescent turn-off sensor based on sulfur-doped graphene QDS in colloidal and film forms was designed for the ultrasensitive detection of carbamate insecticides. The developed fluorescent sensor enabled the detection of carbofuran in a real sample with ppb level sensitivity [110].

Molybdenum disulfide (MoS_2_), an inorganic analog of graphene with a distinctive structure, has recently been envisioned as the next generation 2D layered transition-metal dichalcogenide nanomaterial for highly diversified sensing applications in environmental monitoring [111]. Electrochemical acetylcholinesterase (AChE) biosensor, based on AuNPs-MoS2-reduced graphene oxide/polyimide flexible film (rGO/PI) electrode, has been generated for the detection of the insecticide paraoxon. The study proved a successful fabrication and detection of paraoxon in the linear range 0.005–0.150 μg/mL, a sensitivity of 4.44 μA/μg/mL, and a LOD of 1.4 ng/mL [112].

All these sensors have the potential to be integrated into the sensor network for the sustainable management of agrochemicals delivery, supporting increasing crop productivity.

A lot of work has also been dedicated to the development of IoT-based smart irrigation systems enabling monitoring of crucial parameters, including quantity and quality of waters, characteristics of soil, and weather conditions [113]. Furthermore, in the IoT sector, the Arrowhead technology in the smart agriculture area with smart energy and smart cities was also proposed as an innovative approach to improve interconnectivity and interoperability among different smart devices [114]. Furthermore, the application and deriving advantages of terahertz sensing in the agriculture sector as a faster and reliable technology for the overall monitoring and maintaining leaves’ health was also highlighted in a recent review [115].

## 6. Nanotools for Food Safety and Security

A recent UNFAO estimation showed that, despite some progress, hunger and malnutrition are still crucial challenges and projected difficulties in achieving the Zero Hunger SDG by 2030. The document also discussed the effects of the Covid-19 pandemic on food security and nutrition, highlighting a worsening of the already insecure and unsafe food and nutritional status of the vulnerable segment of both developing and developed countries [116]. In this scenario, developing nations are at higher risk for food shortage and safety, while developed countries should be focused on adopting more rigorous food safety standards and governance and avoid food wastage [117]. Food safety, food security, and healthy nutrition are the key priorities of any food system. Incidental contamination caused by harmful viruses, bacteria, toxins, parasites, and chemicals could determine food poisoning, serious health consequences, and even death in people with a compromised immune system [118]. The source of food contamination can occur anywhere in the supply chain, from production to processing, storage, packing, transportation, and consumption. As such, the availability of tools for early detection, hazard removal, and monitoring should be performed from harvest to consumption to prevent foodborne illness [119].

Nanotechnologies and nanosciences may provide significant sound, sustainable, and resilient solutions to tackle the possible risks derived by food contamination.

Various nanoscale materials/sensors, including metal-organic frameworks (MOFs), graphene oxide (GO), CNTs, molecularly imprinted polymers (MIP), nanozymes, and nanosensors, have been cooperatively used to detect, remediate, and extract unwanted compounds from food sources [120]. These nanoscale materials have also been used as components in devices tailored to the detection of chemical and biological contaminants, allowing the removal from the supply chain of toxic compounds, hence minimizing the distribution of contaminated products to the consumer [121].

In addition to their crucial role in the quality control of food, nanoscale materials are also emerging as pivotal in the food preservation and packing processes [122]. In this regard, various research groups have designed and applied different materials that are compatible with food quality control, preservation, and packaging. In a recent study, an efficient nano-enzymes-based biosensor has been fabricated and employed for the detection of chemical (ions and pesticides residues) and biological contaminants (pathogens and biotoxins) in food [123]. In this context, the synthesis of metal-organic framework (MOFs) and MOFs-based functional materials was revealed as fundamental for the realization of a sensing platform for food safety monitoring and food processing, covering preservation, sanitation, and packaging (Figure 4). Furthermore, extensive research has been conducted in the field of nanostructured emulsions and nanolaminates as delivery systems of AI, including plant-derived antimicrobials and nutraceuticals, as food additives to satisfy consumer demands. The food products containing nanoadditives are novel foods and consequently should get approval for commercialization. Synthesis routes and fine control of physico-chemical parameters have been identified to achieve effective delivery and preservation activity of the functional properties [124].

### Nanomaterials for Food Packaging

Current eating habits, inclined towards the consumption of more natural and fresh products, as well as the need to transport food over long distances in an increasingly connected world, make food packaging a key product in the food production chain. Thus far, plastic materials based on polymers derived from fossil hydrocarbons have played a central role in the design of suitable packaging for different benefits (disposable, thermal, multi-layered packaging, etc.). However, the negative environmental impact of conventional plastics is increasingly drawing the attention of users and authorities, demanding the development of more benign alternatives to the environment [125]. On the other hand, the recent epidemics that shocked the world (in particular, the pandemics caused by the H1N1 and SARS-COV2 viruses), make clear the need for safe packaging, which minimizes the risks of microbial and particularly viral contamination. These conditions drive the development of suitable materials for the preparation of functional and biodegradable packaging with low environmental impact. Packaging should consider the possibility of reusing food waste in a circular economy and of engineering new functional materials with controlled physical and chemical properties for improving food security, in line with SDG2 [6].

Advanced food packaging is designed not only for protection but also to incorporate different ingredients that will provide special benefits (non-toxic antimicrobials, antioxidants, etc.). Functionalized films and coatings based on natural biodegradable polymers (including edible ones) are an environmentally friendly alternative that offers the advantage of increasing the shelf life of many foods (Figure 4). Natural polymers, such as proteins and carbohydrates, show promising physico-chemical properties. However, they must be improved to replace conventional plastics [126].

Natural protein polymers are of great interest due to their biodegradability and the nutritional quality of the protein, which makes it possible to produce edible packages and the possibility of using proteins obtained as a by-product of low commercial value in the manufacture of food. Sodium caseinate is the favorite dairy protein and has been studied by numerous authors [127,128,129]. Films and plastics made from caseinate and a suitable plasticizer have acceptable mechanical properties but have a high commercial value (which goes hand in hand with their food value). Whey concentrate (WPC) has been studied mostly in isolate form (WPI), and the films made from it have poorer properties than caseinate.

To improve the physical properties of protein-based plastic films (and those based on other biopolymers in general), different types of nano-fillers have been used, which, due to their high surface/volume ratio, maximize the effect by minimizing the necessary loading concentration. Good interactions between the filler and the matrix, for example, through hydrogen bonding or dipole interactions, among others, allow the structure of the film to become more resistant, sometimes at the expense of elasticity. If the size of the loading particles is nanometric, the interactions are stronger, achieving good results with a lower content of particles. As an example, the inclusion of nano-fillers of biological origin as reinforcement for WPC or WPI films, such as nanocellulose [130], nanocellulose and chitosan NPs [131], among others. The use of nano-fillers of inorganic origin as reinforcement in protein films, such as TiO_2_, ZnO, and SiO_2_, has also been reported [132], among others.

TiO_2_ is a very interesting alternative as fillers for this type of plastic film, as it has low toxicity, is chemically inert, and has been proven to have antimicrobial activity against *E. coli* and *S. aureus* [133]. Spherical or spheroidal submicron particles have commonly been used.

Zhou et al. and Li et al. [134,135] have studied films prepared with whey protein isolates; Wang and co-workers [136] prepared soy protein isolate films; and Lei et al. [133] have obtained films from a mixture of milk proteins (casein and whey protein isolate). In all cases, the addition of TiO_2_ acted as a mechanical reinforcement and barrier against gases and water vapor. As TiO_2_ has only been used as spherical particles, it is important to study the impact of particles with different geometries since they will most likely affect the mechanical and gas barrier properties in different ways.

ZnO is another alternative as a filler for these films, as it is considered GRAS (generally recognized as safe) by the US Food and Drug Administration (FDA). Due to this characteristic, it can be used in food packaging. ZnO occurs in various morphologies (spheres, rods, needles, tubes, etc.) and offers different structural alternatives and microbiological activity. ZnO has antimicrobial activity (particularly antibacterial), and it is thermally resistant, which gives it an advantage over other organic antibacterials. The antibacterial activity of ZnO present in polymeric films is abundantly documented (see, for example, ZnO in polypropylene: [137]; in PLA: [138]; packaging for food in general: [139]). However, even though the antiviral action of ZnO has been reported, its efficacy, when found in films, is low or null, although the same film presented antibacterial activity [140].

Some authors have included hydrophobic substances in the films obtained from milk proteins or carbohydrates, such as vegetable oils [128], essential oils [141], fatty acids [142], and waxes [143,144] to improve the water vapor barrier properties. The effect was very dependent on the dispersed phase droplet size. The resulting barrier properties were similar between solutions and conventional emulsions but were improved for nanoemulsions. Most of these studies have been carried out using conventional solutions or emulsions without studying the microstructure and its relationship with mechanical properties. Although the use of nanoemulsions, as precursors of this type of film, could improve their physical characteristics by virtue of their high stability and homogeneous droplet size distribution, the films obtained from them have not yet been well studied. The addition of a lipid phase has the additional advantage of dissolving hydrophobic substances with beneficial properties for health or with antimicrobial action. Indeed, the possibility of including antioxidants or fat-soluble vitamins (beta carotenes, vitamin D, omega-3 oils) in the lipid phase, which provides some protection against the oxidizing action of the air and even provides nutraceutical benefits to the food they contain, has beneficial properties. Additionally, the inclusion of essential oils can be mentioned for their antimicrobial [141], and in particular antiviral action [145,146,147]. Essential oils embedded in films may act on viruses before they enter and infect cells, generating an active barrier against the passage of microorganisms.

The incorporation of lipid phases and/or the reinforcement with nano or submicron particles of different shapes modifies the microstructure of the film and consequently its physico-chemical and mechanical properties. These modifications can lead to an improvement or a deterioration of the characteristics of the films. As these are changes produced at the nano or submicron scale, to analyze structural modifications in polymers, it is necessary to use appropriate characterization techniques at that scale, such as small-angle X-ray scattering (SAXS), X-ray microimaging [136,148,149,150,151], and electron microscopy among others.

**Figure 5:**
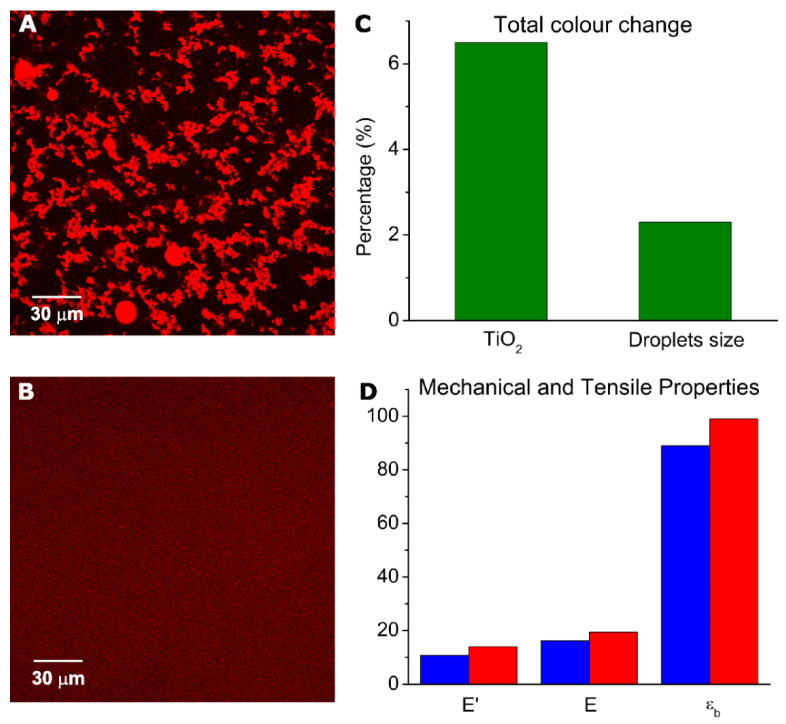
Microstructure of starting systems (left column) and physical properties of the obtained films (right column). Laser scanning confocal microscopy images of emulsions used to prepare films: (**A**) conventional emulsion; (**B**) nanoemulsion. Physical properties: (**C**) total color change due to addition of TiO_2_ or to changes in droplets size (conventional to nano); (**D**) storage modulus (E’), Young modulus (E), and elongation at break (ε_b_). Blue: film from conventional emulsion; red: film from nanoemulsion (reprinted from ref. [152,155,160]).

In a recent study, the use of nanoemulsions of sunflower oil in dispersions of sodium caseinate, using glycerol as plasticizers, led to obtaining stable films without oil exudation. These films were reinforced with spheroidal particles of submicron TiO_2_, which, due to the stability and homogeneity of the precursor dispersions, was uniformly dispersed in the material. These systems were characterized in both their mechanical properties and their microstructure, using advanced material characterization techniques. In this way, the relationship between the microstructure (affected by the presence of TiO_2_ and oil nano-droplets) and the mechanical properties [152,153,154,155,156] were determined. Figure 5 shows, for a selected formulation, the effects of the scale of structural elements of starting systems and the addition of TiO_2_ on total color change and mechanical and tensile properties of whey protein concentrate/corn oil films. The reinforcement with TiO_2_ NPs leads to films more suitable to replace plastic materials. In parallel, investigations were carried out on plastic films obtained from starch reinforced with ZnO in the form of nanobars. The antibacterial action of ZnO and their physical properties, mainly mechanical properties and water vapor permeation, were demonstrated [157,158]. These properties were influenced by the size of the ZnO nanorods. The composites with small nanorods maintained the typical B-V type starch structure, while composites with large nanorods induced the formation of an amorphous structure, preventing starch retrogradation during storage. ZnO NPs combined with oregano essential oil were also used as an antimicrobial in active multilayer films based on polyhydroxyalkanoates (PHAs) with and without high barrier coatings of cellulose nanocrystals (CNCs). The resultant multilayer films were characterized to ascertain their potential in biodegradable food packaging. The films presented high antimicrobial and antioxidant activities and improved the barrier to water and limonene vapors [159].

## 7. Environmental and Health Risks Assessments

The promising role of nanomaterials in supporting the transition towards a more sustainable and resilient agriculture requires a mandatory and adequate risk assessment to avoid negative consequences to the environment and human health [160]. Several studies pointed out unintentional exposure to nanomaterials may cause harmful effects as compared to their bulk analogs [161]. It is reported that most of the innovative nanomaterials are highly mobile, easily dispersible, and very reactive once they are released into the environment, i.e., soil, air, and water, posing a potential threat to human health [162]. Regarding the release, it has been observed some metals, e.g., silver (Ag) and Cu, and metal oxides, e.g., ZnO and iron oxide (FeO), may dissolve quickly, while others, e.g., TiO_2_, silicon dioxide (SiO_2_), CNTs, graphene, are more persistent and may pose potential risks and hazards [163]. The prevailing exposure routes of humans to nanomaterials are inhalation (major exposure route), ingestion, and dermal routes, the last being considered negligible. However, environmental and human health risk assessment associated with the use of nanomaterials is still unclear and debatable [164]. Indeed, identification and characterization of hazards along with exposure assessment are mandatory to characterize the risks and, therefore, to adopt the proper actions for risk management and finally, governance. However, it is not an easy task due to the large variety of exploited nanomaterials, either alone or in combination with the bulk material, differences in doses, application mode and frequency, and geographical area features [165].

Similarly, a responsible and precise risk assessment of agrochemicals needs to be established not only at the organism or population level or in terms of dose-response effects, but also by studying toxicological issues at a cellular and intracellular level on organelles, biomolecules, and macromolecules. Furthermore, the integration of life cycle assessment (LCA) to risk assessment methodologies instead of simply combine their results could provide an estimation of ENMs human health impact within LCA and not as a separate tool for LCA, enabling the ruling out of other non-specific impacts [166].

These methodologies have the potential to guide the processes and guidelines enabling the selection of eco-safe, greener, sustainable, innovative, and convenient nanomaterials for agricultural and environmental applications [167,168].

## 8. Conclusions and Future Prospects

Nanotechnology-based applications include nanofertilizers, nanopesticides, slow-release formulations, nanoremediation, nanosensors, and smart packaging represent disruptive innovations playing a fundamental role in facing the emerging agricultural and environmental challenges mainly related to increased food demand, food safety, and sustainability, enabling the achieving of the 2030 agenda of sustainable development goals connected with agriculture and food security, i.e., 1: No poverty (SDG-1), 2: Zero hunger (SDG-2), 3: Good health and well-being (SDG-3), 4: Industry, innovation, and infrastructure (SDG-9), and 5: Life on land (SDG-15).

Further, nanotechnology-based innovations are projected to transform the current conventional agricultural system into a highly efficient, resilient, and sustainable agricultural system. However, it should be emphasized that the nano-based innovations and strategies are still in the growing phase, and more efforts are needed to design eco-safe, cost-effective, more stable, efficient, and multifunctional ENMs. Similarly, the risk assessment procedures are also still under development, being novel ENM materials.

Currently, the applications of ENMs are mostly under controlled laboratory conditions, where the release behavior of nutrients from coated materials to the target could be different compared to natural conditions. Therefore, large in-field experimentations based on scientific information deriving by controlled laboratory conditions are highly recommended to get more in-depth insights on the release behavior of formulated ENMs. Likewise, the agronomic effectiveness of formulated ENMs is still not well documented, requiring extensive research for a variety of crops, including vegetables and field crops, on different soils.

To bring the field of agro-based nanotechnology forward, it is necessary to strengthen research collaborations among universities, research institutes, key research laboratories, and industries for the development, characterization, and application of novel ENMs in the agricultural system. It should also be noted that in many countries, especially in developing countries, the transfer of knowledge and innovative technologies to farmers is often lacking, impairing their capability to enhance productivity and income, and it should be kept in priority.

Finally, full accessibility to scientific data and statistics will play a pivotal role in supporting stakeholders and policymakers in developing proper governance in every country.

## Figures and Tables

**Table 1:** Recent innovations on slow-release pesticides.

Nanopesticides	Remarks	Ref.
**Chitosan Capped-Cu nano-biocomposites** AI: Cu	Novel copper nano-biocomposites have been effectively prepared by utilizing chitosan, a naturally occurring polymer, and an organic source of ascorbic acid. The prepared biocomposites revealed remarkable antifungal and antibacterial activity against notorious agricultural plant pathogens, viz., *Fusarium* sp., *Aspergillus* sp., *Alternaria alternata*, *Pythium* sp., and *Bacillus cereus*, on poisoned food. Moreover, the as-prepared biocomposites revealed are non-toxic, biodegradable, and safe for ecosystem and human health.	[20]
**Kocide^®^3000** **Lab-made Cu(OH)_2_** AI: CuOH_2_; Cu	The trophic transfer of Cu from tomato plants (*Solanum lycopersicum*) to tobacco hornworms (*Manduca sexta*) was investigated following the application of the commercially available fungicide Kocide^®^3000 containing Cu(OH)_2_ nanoneedles as its active ingredient and compared with laboratory-synthesized copper hydroxide nanoneedles nCu(OH)_2_. Obtained data revealed a significant difference in the accumulation and toxicity between lab-based synthesized CuOH_2_ and commercially available Kocide^®^3000. The difference in their toxicity and accumulation and elimination dynamics were found to be correlated with the solubility of the materials in the exposure suspensions.	[21]
**Kocide^®^3000** AI: CuOH_2;_ Cu	The study revealed the effects of Kocide^®^3000 on soil microbial communities’ function, structure, and abundance over 90 days, at single and seasonal agricultural application doses, in the presence and absence of an edaphic organism (the isopod *Porcellionides pruinosus*). The results indicated Kocide^®^3000 might affect soil microbial communities, potentially changing the soil’s ecological role. On the other hand, the presence of invertebrates in soils may mitigate this effect, even if further studies are needed to confirm these results.	[24]
**Biochar-based hydrogel microspheres** AI: Gentian violet (GV)	Biochar-based hydrogel microspheres were successfully manufactured to construct a pH and ion strength dual-stimulus responsively controlled-release system to host the gentian violet (GV) hydrophilic insecticide. The encapsulation of GV in a 3D matrix guarantees its controlled release. The GV pesticide carrier had nearly no harmful effect on cell proliferation of zebrafish embryos, indicating biosafety.	[28]
**Water-dispersible difunctional NPs** AI: Lambda-cyhalothrin	Water-dispersible difunctional NPs have been prepared utilizing FNP, where self-assembling amphiphilic block copolymers were used to encapsulate the highly hydrophobic model pesticide, Lambda-cyhalothrin, and the fluorescent dye Nile Red. The IVIS imaging and confocal laser scanning microscopy analyses showed that the resulting difunctional nanopesticide particles could allow accurate in situ trackings of the pesticide on the leaf surface while effectively avoiding interference from chlorophyll autofluorescence. The insecticidal activity and stability of the difunctional NPs suspension were high.	[32]
**Thiamethoxam (TMX) loaded CNCs** AI: TMX	By using the solvent evaporation approach employing cellulose nanocrystals (CNCs) as the carrier, a novel, nanoformulation of thiamethoxam (TMX) was created. The drug release behavior and bioassays analyses indicated a controlled release of TMX from TMX-loaded CNCs, and improved insecticidal activity of TMX-CNCs against *Phenacoccus solenopsis* compared to the commercial formulation.	[37]
**Chitosan-based fosetyl-Al nanocrystal** AI: fosetyl-Al	The ultrasonication-assisted synthesis of water-dispersible nanocrystals was used to generate a novel formulation of systemic fungicide fosetyl-Al. Chitosan was used as a coating agent to achieve a synergistic antibacterial action between the biopolymer and the fungicide. It was reported that chitosan-based fosetyl-Al nanocrystal proved to be more stable and less harmful compared to conventional formulations. The nanoformulation exhibited remarkable antibacterial activity.	[38]
**Bacterial synthesized ZnO-NPs** AI: ZnO-NPs	ZnO-NPs were prepared by using a culture supernatant of native *Bacillus cereus* RNT6 strain, which was taxonomically recognized by 16S rRNA gene analysis. The biogenic ZnO-NPs showed significant antibacterial activity against *B. glumae* and *B. gladioli* to control diseases in rice.	[39]
**Green nanostructured pesticide** AI: chitosan hydrochloride	A green nanostructured pesticide consisting of chitosan hydrochloride as the active ingredient, cellulose nanocrystals as a nanocarrier, and starch as excipient was synthesized to control tomato bacterial speck disease. The as-prepared pesticide revealed promising inhibitory activity on *Pseudomonas syringae* pv. *Tomato*.	[40]
**CuO nanoparticle on the surface of reduced graphene oxide** AI: CuO	A CuO nanoparticle decorated on the surface of reduced graphene oxide rGO-CuO NPs was successfully prepared. The rGO-CuO NPs showed a promising antifungal effect against *Fusarium oxysporum* and were recognized as a novel eco-safe and cost-effective nanopesticide towards sustainable crop protection.	[41]

**Table 2:** Recent innovations related to CRFs/materials. Encapsulating material: Em.

CRFs/Materials	Remarks	Ref.
**Biochar-based slow-release fertilizer (BSRFs)** Em: Biochar and bentonite AI: K_3_PO_4_)	Keeping in view the promising properties of biochar as a soil amendment and a potential carrier for the slow-release of fertilizers, a biochar-based slow-release fertilizer (BSRFs) was synthesized by co-pyrolysis of corn straw, nutrients (K_3_PO_4_), and bentonite under microwave irradiation. The results highlighted that the presence of clay mineral (bentonite) in the synthesis process is beneficial to improve the controlled-release activity of BSRFs. The as-prepared BSRFs are cost-effective, eco-safe, and have high utilization efficiency.	[55]
**Dual-release engineered phosphate fertilizers** Em: Graphene oxide AI: mono-ammonium phosphate	A unique strategy was adopted to prepared engineered phosphate fertilizer with dual-release characteristics, i.e., fast and slowly soluble phosphorous by compaction method. The composition was made by mono-ammonium phosphate (MAP) acting as a highly soluble phosphorus nutrient source and a commercially available slow-release phosphorous, such as struvite (Str) or P-loaded graphene oxide. Briefly, the results highlighted the better performance of the dual-release phosphorous fertilizers as compared to MAP and Str.	[56]
**Zein-coated porous carboxymethyl starch (PCS)** Em: Zein (A natural polymer) AI: P and Fe	Zein-coated porous carboxymethyl starch (PCS) absorbent was prepared to enhance the utilization efficiency of phosphorous and simultaneously supply available Fe. The main objective was to develop PCS-Fe-P/Zein fertilizer. The reason for the inclusion of Fe (a microelement for chlorophyll synthesis) in the fertilizer was to act as a bridge between PCS and phosphate ions. Briefly, the results highlighted that the cumulative release of phosphorus in water was 18% in 30 days of duration. Moreover, the fertilizing effect of PCS-Fe-P/Zein was tested on soybean in a pot experiment and the utilization efficiency and uptake of phosphorous was 68%. The overall results indicated that PCS-Fe-P/Zein fertilizer has potential and sustainable application in agriculture.	[57]
**Poly(tannic acid)-coated urea fertilizer** Em: Tannic Acid AI: Urea	A novel green coating material, “a poly (tannic acid) (PTA)-coated fertilizers with urea prills as the core”, was prepared through a novel, simple method, “spout fluidized bed”, by using a natural polyphenol tannic acid (TA). The experimental results both in water and soil presented that the release rate of nitrogen from the as-prepared material was much lower than that from raw urea. The method developed in this paper is eco-safe for the preparation of SRFs has high potential in sustainable agriculture.	[58]
**Controlled-release potassium chloride(CRK)** Em: KCl AI: potassium (CRK)	A controlled release potassium chloride (CRK) was mixed with traditional KCl fertilizers in a 1:1 ratio to improve the utilization efficiency of potassium and lower the manufacturing cost. Based on experimental findings, the as-prepared CRK mixed with KCl was recommended for the delayed release of potassium to enhance soil fertility, increase crop yield on a sustainable basis, and maximize farmer’s income.	[59]
**Degradable polyester/urea inclusion complex** Em: Polyester AI: Urea	A facile solvent-free approach has been adopted to develop a novel environmentally degradable polyester/urea inclusion complex by one-step blending as slow-release fertilizers. Briefly, the results revealed that the polyester/urea inclusion complex showed a good slow-release rate compared to raw urea.	[60]
**Sulfur-containing urea SRFs** Em: Poly (eugenol sulfone) AI: Sulfur (S)	A novel slow-release sulfur-containing urea fertilizer with good biodegradation performance was synthesized by coating with sustainable poly (eugenol sulfone) derived from renewable eugenol and SO_2_ via simple free radical polymerization under mild conditions. The obtained results based on a set of designed experiments proved that the sulfur-containing urea fertilizer showed outstanding slow-release characteristics, biodegradation features and provides a new route for S-cycling.	[61]
**Composites of biopolymers and ZnO NPs** Em: Biopolymers AI: Zinc (Zn)	To increase the utilization efficiency of Zn micronutrient for plant growth, a composite composed of biopolymers (microcrystalline cellulose, chitosan, and alginate) and ZnO NPs (4–65% Zn w/w) was synthesized. The potential controlled release kinetics of Zn from the as-prepared composites in comparison to conventionally used Zn salts was tested in four different types of agricultural soils. Briefly, the results indicated that ZnO-biopolymers maintained a better constant supply of CaCl_2_-extractable Zn compared to all other treatments. Overall, the ZnO-alginate beads synthesized through crosslinking with CaCl_2_ showed the slowest controlled release kinetics of Zn.	[62]
**Biodegradable urea-formaldehyde/poly(butylene succinate) and its ternary nanocomposite**	Urea-formaldehyde-based or polybutylene succinate-based composites and nanocomposite used as a slow-release fertilizer in agriculture provide good feasibility for a large-scale application.	[63]
**Nano-Delivery systems with Nature-Derived Polymers**	Provided detailed scientific literature information on the advances in the innovations of nano-delivery systems using nature-derived polymers for agri-food applications.	[64]

**Table 3:** Latest innovations regarding nano-enabled materials for soil remediation.

Nanomaterials	Remarks	Ref.
**CaAl-layered double hydroxide (CaAl-LDH)**	The efficacy of CaAl-layered double hydroxide (CaAl-LDH) as an efficient stabilizer in cadmium-contaminated soil has been investigated. Due to its 96.9% immobilization efficiency for cadmium in contaminated soil, this material was recommended as a promising competitive candidate for facile remediation of Cd-contaminated soils.	[81]
**Micro zerovalent iron (ZVI) and nano zerovalent iron (nZVI)**	The capability of ZVI and nZVI to efficiently immobilize metals and metalloids, i.e., As, Cu, Cr, Zn, and Pb, were compared by analyzing two different contaminated soils. nZVI revealed the best performance for all the tested species. Analyses on the long-term stability of metal (loid) by thermal oxidation tests revealed good retention of As and Cu and significant desorption of Pb. For the long-term stability of metal (loid)s in soil, the exploitation of micro-Fe over nano-Fe has been recommended due to the slow oxidation of the former.	[82]
**Low-cost Fe/Al-based materials**	The use of three low-cost Fe/Al-based materials, including red soil (RS), sponge iron filters (SIF), and Al-based water treatment sludge (WTS), as amendments to remediate arsenic-contaminated soils under anoxic condition, was analyzed. SIF was revealed to be the more promising material in comparison with the other two to remediate the As-contaminated soil.	[83]
**A thiol-modified rice straw biochar (RS)**	A novel thiol-modified RS was synthesized by esterification with β-mercaptoethanol for the remediation of Cd^2+^ and Pb^2+^ contaminated soils. RS selectively adsorbed Cd^2+^ over Pb^2+^ and reduced Cd availability by up to 40% while enabling limited immobilization of Pb. RS revealed an effective amendment for remediation of soil pollution.	[84]
**Nanoscale ferrous sulfide**	The efficiency, stability, and feasibility of using sodium carboxymethyl cellulose-stabilized nanoscale ferrous sulfide (CMC-nFeS) for the immobilization of Cr (VI) in contaminated soil were demonstrated, along with the reaction mechanism between CMC-nFeS and Cr (VI) in a neutral environment.	[85]
**Zeolite-supported nanoscale zero-valent iron (Z-NZVI)**	This study provides detailed information on the encapsulation mechanisms of heavy metals/loids (Cd, Pd, and As) and the ecological risks of Z-NZVI in real farmland soils. The secondary mineralization of Z-NZVI bonded metal (loid)s, and improved soil quality afford its use for the long-term remediation of metals/loids contaminated soils without significant ecotoxicological risks.	[86]
**Zinc oxide NPs (ZNONPs)**	The potential of ZNO-NPs nanofertilizer to simultaneously reduce both As and Cd and nourishing rice tissues was tested in a greenhouse. The results highlight the ZNO-NPs nanofertilizer potential to reduce As and Cd in rice paddies, strengthening the key role of, and providing new insights into, the nanotechnology application in agriculture.	[87]
**Flower-like magnetic MoS_2_ nanohybrid**	The synthesis, characterization, and application of flower-like molybdenum disulfide decorated with iron oxide NPs (MoS_2_/Fe_3_O_4_) by a two-step hydrothermal method for the removal of Hg(II) and Pb(II) in the aqueous solution in the soil were described. Considerations on the facile preparation route, easy operation, and high removal efficiency of sulfide-based nanohybrid laid a foundation for the development of promising adsorbent strategies to remove heavy metals from wastewater and soils.	[88]
**Biochar/iron (BC/Fe) composites**	Biochar/iron (BC/Fe) composites revealed the interdependent effect of BC and Fe in soil and water remediation. The effect was owing to the mutual combination of Fe (adsorption, reduction, and oxidation) and BC (high surface area, rich functional groups, and high electron transfer efficiency).	[89]
**Biogenic manganese oxide (BMO) materials**	BMO was successfully used to stabilize arsenic in contaminated soil. The results also suggested BMO is a more efficient, cost-effective, and eco-friendly material compared to manganese oxide for remediation of arsenic. The study revealed a positive effect on the soil bacterial community biodiversity.	[90]
